# Prediction of carcass weight using the morphometry of ankle bones in hair goats

**DOI:** 10.1002/vms3.1544

**Published:** 2024-07-17

**Authors:** Yasemin Üstündağ, Mehmet Kartal

**Affiliations:** ^1^ Veterinary Faculty, Department of Anatomy Dokuz Eylül University İzmir Turkey; ^2^ Health Sciences Faculty İstanbul Gelişim University İstanbul Turkey

**Keywords:** calcaneus, carcass weight, hair goat, regression, talus

## Abstract

**Bacground:**

Morphologic measurements such as body lenght, wither height, heart girth, chest width, body leght, cannon‐bone circumference is used to predict carcass weight. For this purpose, estimating carcass weight with measurements of key bones such as ankle bones, which play a significant role in the balance distribution of body weight, seems possible.

**Objectives:**

The aim of this study is to create new regression models for effective carcass weight estimation by using the morphometric data of the talus and calcaneus bones of hair goats.

**Methods:**

Study materials consisted of talus and calcaneus bones obtained from abattoir products of hair goat kids (12–18 months old, 20 female and 20 male) and adult hair goats (36–48 months old, 20 female and 20 male). Morphometric measurements of the talus and calcaneus of each animal were taken by a digital caliper. Using the morphometric measurements, an index and a factor were calculated for each bone. Regression analysis and correlations were examined in IBM SPSS 21 programme.

**Results:**

As a result, statistical analysis of GLc, GLt, Bd, Calfactor and Talfactor were statistically significant on predicting carcass weight.

**Conclusion:**

Specific anatomical structures, such as certain bone measurements, such as talus and calnaneus could serve as indicators of growth performance and also carcass weight performance. In addition new anatomical factors and indices may be produced and new regression methods may be applied with these new parameters to predict carcass weight.

## Graphical abstract

The study investigated the relationship between age, sex and carcass weight in hair goats through morphometric measurements of the talus and calcaneus bones. Significant correlations were found between bone measurements (GLc and GLt) and carcass weight, with regression models predicting carcass weight with 85% accuracy. Age significantly influenced morphometric measurements, whereas sex did not significantly affect both morphometric measurements and predicting carcass weight.

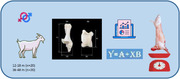

## 1 INTRODUCTION

The domestic goat, known scientifically as *Capra hircus*, is a member of the *Capra* genus within the Bovidae family. It holds the distinction of being one of the earliest domesticated animal species, alongside sheep. From the dawn of human civilization, goats have been raised for their meat, milk, leather and hair (including mohair, fibre and cashmere). Their ability to adapt to various environmental and breeding conditions, as well as their resistance to numerous diseases, makes them a popular choice for rearing (Demiraslan et al., 2021). The significance of goats in human life is expected to grow even further in the future, as researchers. Goat farming not only fulfils the demand for animal protein, a crucial component of human nutrition, but also generates employment opportunities in the Aegean, Mediterranean and Black Sea regions, especially in barren high‐altitude regions, given the challenging geographical conditions in Turkey, thereby contributing to income generation for people in rural areas. Therefore, it is essential to practice mindful farming to maximize the benefits derived from goats (Tozlu Çelik & Oflaz, 2017). According to goat farming in Turkey, the Turkish hair goat, a native goat breed, is the most populous and dominant breed in the Black Sea region, having adverse conditions, comprising approximately 95% of the 6 million Turkish goat population and having a major impact on goat meat production (Cam et al., 2010; Simsek & Bayraktar, 2006; Yılmaz et al., 2013). The hair goat possesses a strong ability to adapt to harsh environmental conditions and has a robust physiological defence mechanism against diseases. However, it should be noted that despite their popularity, they are subject to a small population in order to protect the forest areas in Turkey (Koyuncu et al., 2007).

Characteristics related to growth and development can serve as criteria for early selection. The primary objective of farming is to extract the maximum value from each animal in the shortest possible time. In this context, conscious farming should be practiced to derive maximum benefit from goats (Tozlu Çelik & Oflaz, 2017). Growth, defined as an increase in cell and tissue mass and volume, varies based on genotype and environmental conditions and continues until a certain age, as stated by Chacon et al. (2011). It has been observed that certain macro‐environmental factors, such as genotype, gender, type of birth, feeding environment, age, year of birth and the age of the mother, influence body measurements in animals (Chacon et al., 2011).

Morphologic measurements, such as body length, wither height, heart girth, chest width and rump height, are among the most important parameters used to determine the relationship between body size and weight (Afolayan et al., 2006; Lambe et al., 2008; Ofori et al., 2020; Quaresma et al., 2019). However, some individuals have denser bones and larger frames. Therefore, body measurements by themselves may not offer factual data about body weight. Moreover, considering livestock carcass weight is an important factor in determining the amount of meat that can be obtained from slaughtered animals, which is dependent on the animal's live weight before slaughter and yield. Besides, chest width, chest depth, body length and cannon‐bone circumference are used to predict carcass weight (Ateş & Akbaş, 2022). For this purpose, estimating carcass weight with measurements of key bones such as ankle bones, which play a significant role in the balanced distribution of body weight, seems possible (Lambe et al., 2008; Quaresma et al., 2019; Şireli, 2018).

In ruminants, ankle bones are arranged in three rows: the proximal, middle and distal. The proximal row has two bones: the calcaneus (c) and talus (t). The middle row contains one bone that represents the combined central and fourth tarsal bones, called the centroquartal bone. The distal row consists of two bones, fused second and third tarsal bones and a singular first tarsal bone (Getty, 1975; Liebich et al., 2004; Schimming et al., 2015).

Proximal row is responsible for forming the hock joint, which provides stability and support to hind legs as well as allowing for movement and flexibility. It consists of two bones: talus and calcaneus. The talus is located at the top of the ankle joint and provides a connection between the lower part of the leg and the foot. This bone transfers body weight from the ankle to the foot. Therefore, it has a significant impact on the distribution and balance of body weight. The calcaneus, or heel bone, lies lateral and plantar to the talus and provides the osseous base of the point of the hock. It is the main structure that carries the weight of the body. It absorbs the shocks that occur when walking, running or jumping. It also affects the distribution of body weight on the foot (Getty, 1975; Liebich et al., 2004; Nickel et al., 1977).

The best method of weighing animals without a scale is to regress body weight on certain body measurements, which also could be used to predict carcass weight and meat quality (Bassano et al., 2003; Cam et al., 2010; Lambe et al., 2008; Simsek & Bayraktar, 2006). Considering the facts mentioned above, the present study was planned to assess the carcass weight of hair goats by using ankle bone measurements based on the talus and calcaneus and formulate bone indices and bone factors to estimate the correlation between age and sex of these animals. On the basis of one or more morphometric measurements, by using simple regression, which represents a practical method of predicting the carcass weight of goats for farmers, the best prediction equations could be developed.

## 2 MATERIALS AND METHODS

In the present study, study materials consisted of talus and calcaneus bones obtained from abattoir products of hair goat kids (12–18 months old, 20 females and 20 males) and adult hair goats (36–48 months old, 20 females and 20 males). Turkish people's meat preference was taken into consideration while determining the age range of the animals. Age and sex of these animals were recorded before slaughter, and carcass weights were taken after slaughter. Right and left talus and calcaneus were subjected to maceration by the boiling method (Onar & Kahvecioğlu, 1999).

Morphometric measurements of the talus and calcaneus of each animal were taken using a calibrated electronic digital calliper with a sensitivity of 0.01 mm, an accuracy of ±0.01 mm (<100 mm) and a repeatability of 0.01 mm. The calliper was measured against a set of six known block values. Measured values obtained from the calliper were recorded to the nearest 0.25 mm for three separate measurements from each block to check accuracy and repeatability. The average measured value was checked against the block value to verify the accuracy. Measurements were based on von den Driesch and Boessneck (1974).

Morphometric measurements:

Talus:

GLt: Greatest length of the lateral half
Bd: Greatest breadth of the distal end

Calcaneus:

GLc: Greatest length
GB: Greatest breadth

The talus and calcaneus mean and standard deviation (SD) values were calculated. The normality of the value distribution of the measurements was checked using the Shapiro–Wilk test (George & Mallery, 2010). An independent sample *t* test was used to check the significance of the difference between the mean values of both sexes and between the mean values of the right and left bones.

Using the morphometric measurements, an index was calculated for each bone. These index values were given as talindex for talus and calindex for calcaneus:

Index = GB(Bd)/GL

All morphometric measurements of the bones were used to check for homotypic variation between right and left bones. Regression analysis and factor calculation were performed to reveal the relationship among GLt, GLc, Bd and GB and carcass weight and to be used in carcass weight estimations. Correlation analysis was performed to reveal correlation between the carcass weight and morphometric values. SPSS 21.0 programme (Version 29.0, SPSS Inc.) was used for statistical analysis.

Apart from regression analysis, talus and calcaneus GL measurements were rationed to carcass value to obtain multipliers for both talus (astfactor) and calcaneus (Calfactor). The obtained factors were calculated with the following formulation:

Talfactor = Carcass weight (kg)/GLt
Calfactor = Carcass weight (kg)/GLc

In order to estimate the calcaneus and talus GL values depending on a value, the ratio of calcaneus GL and talus GL was calculated. Thus, a multiplier (GLc/GLt) was obtained for the other GL estimation, depending on a known GL value from the calcaneus or talus.

The least squares means (LSM) test was applied to see whether there was an effect of sex and age on the carcass weight and morphometric measurements, index and factor values obtained.

The carcass weight regression equation was obtained using indexes, which were statistically significant. Variables were formulated in the following equation (Katherina & Sudirati, 2020):

*y* = *a* + *bx*

where *y* is the dependent variable (carcass weight), *a* is the constant term, *b* is the coefficient and *x* is the independent variable (morphometric measurements, index and factor values).

The following formulas were used for the coefficient and constant term for each separate equation:

$$a=\bar{y}-b\bar{x}$$

$$b=\frac{\sum xy-n\bar{x}\bar{y}}{\sum x^{2}-n\bar{x}\bar{x}}\;$$

The statistical data obtained from the results are presented in tables, and the World Association of Veterinary Anatomists (2017) was used as the basis for writing the study.

## 3 RESULTS

A total of five parameters, such as carcass weight, GLc, GB, GLt and Bd, were measured, and four calculations, such as Calfactor, Talfactor, calindex and talindex, were designed. Descriptive statistics were examined. All of the values obtained from descriptive statistics were distributed normally according to the Shapiro–Wilk test (*p* > 0.05).

Carcass weights of the goats used in the study according to both ages and sexes are given in Table 1. The results of the independent sample *t* test are given in Tables 2 and 3. According to tables, there is a significant relationship between age and carcass weight in both young (12–18 months) and adult (36–48 months) goat groups at the *p* < 0.001 level. The effect of age on young and adult goats (on GLc) is significant on both the right and left sides (*p* = 0.04 and *p* = 0.03 at *p* < 0.05 level consecutively); on GLt, it is significant on the right and left sides at *p* < 0.001 level and *p* = 0.003 at *p *< 0.05 level consecutively. In terms of Calfactors and Talfactors for both young and adult goats, there is a significant relationship between age and carcass weight at the *p* < 0.001 level. Although there is a significant relationship between age and only left calindex of adult goat group at the *p* < 0.001 level, there is a significant relationship between age and right talindex of both age groups significantly at *p* < 0.001 level and *p* = 0.04 at *p* < 0.05 level consecutively. The other parameters are not statistically significant at *p *< 0.05 level.

**TABLE 1:** Carcass weight by sex and age (kg).

Age (months)	Sex	Mean	*N*	SD	Minimum	Maximum
12–18	Female	24.65	20	2.25	22.0	31.0
	Male	30.20	20	2.30	26	34.0
36–48	Female	26.10	20	3.27	23.0	34.0
	Male	31.15	20	3.63	26.0	37.0
12–18	Total	27.42^*^	40	3.60	22.0	34.0
36–48	Total	28.62^*^	40	4.26	23.0	37.0

* Statistically significant at *p* < 0.001 level.

**TABLE 2:** Independent sample *t* test results for morphometric measurements.

Morphometric measurements	Age (months)	*t* Test	
		*t*	*p*
Carcass weight	12–18	−7.874	<0.001^**^
	36–48	−4.770	<0.001^**^
GLc – right	12–18	2.127	0.04^*^
	36–48	1.435	0.08
GLc – left	12–18	2.161	0.03^*^
	36–48	1.433	0.08
GB – right	12–18	0.443	0.66
	36–48	1.158	0.25
GB – left	12–18	0.188	0.85
	36–48	0.603	0.27
GLt – right	12–18	−4.094	<0.001^**^
	36–48	0.238	0.40
GLt – left	12–18	−2.928	0.003^*^
	36–48	1.817	0.07
BD – right	12–18	0.423	0.33
	36–48	0.972	0.16
BD – left	12–18	−0.732	0.23
	36–48	1.111	0.13

* Statistically significant at *p* < 0.05 level.

** Statistically significant at *p* < 0.001 level.

**TABLE 3:** Independent sample *t* test results for factors and indexes.

Factors and indexes	Age (months)	*t* Test	
		*t*	*p*
Calfactor – right	12–18	−7.132	<0.001^**^
	36–48	−5.362	<0.001^**^
Calfactor – left	12–18	−7.25	<0.001^**^
	36–48	−5362	<0.001^**^
Talfactor – right	12–18	−4.045	<0.001^**^
	36–48	−4.532	<0.001^**^
Talfactor – left	12–18	−4.896	<0.001^**^
	36–48	−5.466	<0.001^**^
Calindex – right	12–18	−0.131	0.44
	36–48	0.610	0.27
Calindex – left	12–18	−1.315	0.09
	36–48	−5.201	<0.001^**^
Talindex – right	12–18	3.429	<0.001^**^
	36–48	1.727	0.04^*^
Talindex – left	12–18	1.223	0.11
	36–48	0.189	0.42

* Statistically significant at *p* < 0.05 level.

** Statistically significant at *p* < 0.001 level.

The LSM test did not reveal any significant impact of sex on the weight of the carcass. This effect was mentioned in the average values for sex in Table 1. However, the differences between these average values were not statistically significant at *p* < 0.05 level.

In the LSM test on calcaneus values, the effect of age was observed, and results are given in Table 4. The effect of age was observed on GLc, GB and Calfactor values in both young and adult individuals. The difference between the mean values of GLc, GB and Calfactor in young female individuals was significant at *p* < 0.05 level, and *R* ^2^ values were 0.309, 0.258 and 0.856. In young male individuals, GB was significant at *p* < 0.05 at level, *R* ^2^ = 0.358 and Calfactor was significant at *p* < 0.001 level, *R* ^2^ = 0.768. The difference between the mean values of GLc and GB in adult female individuals was significant at *p* < 0.05 level; *R* ^2^ values were 0.402 and 0.309. In adult male individuals, only Calfactor is statistically significant at *p* < 0.001, *R* ^2^ = 0.841. The effect of age was non‐significant on Calindex of all of the individuals at *p* < 0.05 level.

**TABLE 4:** Morphometric values of calcaneus (mm), factor and index values of calcaneus and least squares means (LSM) test results on calcaneus values.

Right calcaneus	Statistical	GLc	GB	Calfactor	Calindex	Left calcaneus	Statistical	GLc	GB	Calfactor	Calindex
Female (12–18 months)	Mean	68.54^*^	22.33^*^	0.35^*^	5.68	Female (12–18 months)	Mean	68.52^*^	22.23^*^	0.35^*^	5.68
	*N*	20	20	20	20		*N*	20	20	20	20
	SD	2.66	1.43	0.03	0.60		SD	2.77	1.00	0.03	0.62
	Minimum	64.02	19.67	0.30	4.52		Minimum	64.73	20.18	0.30	4.69
	Maximum	73.01	24.54	0.46	6.52		Maximum	73.96	23.89	0.46	6.65
Male (12–18 months)	Mean	70.41	22.13^*^	0.44^**^	5.70	Male (12–18 months)	Mean	70.12	22.26^*^	0.44^**^	0.62
	*N*	20	20	20	20		*N*	20	20	20	20
	SD	2.89	1.30	0.04	0.67		SD	2.54	1.45	0.03	5.93
	Minimum	65.23	19.98	0.37	4.55		Minimum	64.89	19.12	0.36	4.74
	Maximum	74.68	24.62	0.51	6.69		Maximum	73.19	25.02	0.51	7.07
Female (36–48 months)	Mean	73.31^*^	22.77^*^	0.34	5.52	Female (36–48 months)	Mean	73.40^*^	23.06^*^	0.34	5.52
	*N*	20	20	20	20		*N*	20	20	20	20
	SD	5.42	1.79	0.03	0.54		SD	5.34	1.89	0.03	0.65
	Minimum	65.75	19.76	0.28	4.41		Minimum	65.75	20.46	0.28	4.31
	Maximum	85.75	26.03	0.43	6.50		Maximum	86.66	26.94	0.43	6.81
Male (36–48 months)	Mean	76.27	23.54	0.42^**^	5.40	Male (36–48 months)	Mean	76.21	23.41	0.42^**^	5.43
	*N*	20	20	20	20		*N*	20	20	20	20
	SD	7.47	2.34	0.05	0.65		SD	6.96	1.75	0.06	0.54
	Minimum	65.75	20.46	0.30	4.31		Minimum	66.00	20.23	0.30	4.41
	Maximum	92.29	29.19	0.54	6.81		Maximum	89.59	26.12	0.54	6.50

* Statistically significant at *p* < 0.05 level.

** Statistically significant at *p* < 0.001 level.

In the LSM test on the talus values, the effect of age was observed, and results are given in Table 5. The effect of age was observed on GLt, Bd and Talfactor values in both young and adult individuals. The difference between the mean values of GLt and Talfactor in young female individuals was significant at *p* < 0.01 level, and *R* ^2^ values were 0.396 and 0.885. Bd was significant at *p* < 0.05 at level, *R* ^2^ = 0.383, and Talfactor was significant at *p* < 0.001, *R* ^2^ = 0.723 in young male individuals. The difference between the mean values of Bd was significant at *p* < 0.05 level; *R* ^2^ = 0.402; GLt and Talfactor were significant at *p* < 0.001; *R* ^2^ values were 0.727 and 0.826 in adult female individuals. In adult male individuals, only Talfactor was statistically significant at *p* < 0.001; *R* ^2^ = 0.856. The effect of age was non‐significant on Talindex of all of the groups at *p* < 0.05 level.

**TABLE 5:** Morphometric values of talus (mm), factor and index values of talus and least squares means (LSM) test result on talus values.

Right talus	Statistical	GLt	Bd	Talfactor	Talindex	Left Talus	Statistical	GLt	Bd	Talfactor	Talindex
Female (12–18 months)	Mean	36.11^**^	17.68	0.68^**^	9.95	Female (12–18 months)	Mean	36.78	17.79	0.67^**^	10.44
	*N*	20	20	20	20		*N*	20	20	20	20
	SD	2.79	1.93	0.06	1.09		SD	2.50	1.69	0.06	0.95
	Minimum	32.04	13.79	0.58	7.82		Minimum	32.81	14.18	0.57	8.52
	Maximum	41.64	21.16	0.83	11.71		Maximum	41.26	21.46	0.80	11.64
Male (12–18 months)	Mean	39.30	17.94^*^	0.77^**^	11.08	Male (12–18 months)	Mean	38.94	18.30^*^	0.77^**^	10.82
	*N*	20	20	20	20		*N*	20	20	20	20
	SD	2.07	1.94	0.06	0.98		SD	2.15	1.83	0.07	1.00
	Minimum	33.51	14.18	0.67	9.00		Minimum	33.34	14.91	0.66	9.20
	Maximum	42.72	21.27	0.90	13.04		Maximum	41.89	21.27	0.93	13.39
Female (36–48 months)	Mean	37.27^**^	17.37^*^	0.69^**^	10.62	Female (36–48 months)	Mean	36.47	17.30	0.68^**^	10.94
	*N*	20	20	20	20		*N*	20	20	20	20
	SD	2.97	1.69	0.06	1.08		SD	2.82	1.66	0.07	1.08
	Minimum	32.56	13.79	0.59	8.67		Minimum	32.81	13.79	0.54	8.67
	Maximum	43.72	21.27	0.86	12.75		Maximum	43.84	20.39	0.84	12.75
Male (36–48 months)	Mean	37.50	17.89	0.84^**^	11.21	Male (36–48 months)	Mean	38.13	17.94	0.85^**^	11.01
	*N*	20	20	20	20		*N*	20	20	20	20
	SD	3.05	1.69	0.12	1.11		SD	2.96	1.94	0.12	1.21
	Minimum	33.04	14.18	0.58	8.78		Minimum	33.04	14.18	0.58	8.78
	Maximum	43.72	21.46	1.13	12.87		Maximum	44.29	21.27	1.11	12.87

* Statistically significant at *p* < 0.05 level.

** Statistically significant at *p* < 0.001 level.

When the ratio of calcaneus GL and talus GL was calculated, an average value of 1.75 for young female individuals, 1.93 for young male individuals, 1.97 for adult female individuals and 2.02 for adult male individuals were obtained (Table 6). The LSM test on this value resulted that age had no effect in adult individuals. But in young individuals, it is statistically significant at *p* < 0.001 level and *R* ^2^ = 0.707. These data were useful for estimating the GL of one of the two bones relative to the GL of the other, especially in young individuals.

**TABLE 6:** Calcaneus GL/talus GL ratios.

Age	Sex	Mean	*N*	SD	Minimum	Maximum
12–18 months	Female‐R	1.74^**^	20	0.11	1.57	2.05
	Female‐L	1.76^**^	20	0.11	1.60	2.05
	Male‐R	1.96^**^	20	0.18	1.58	2.28
	Male‐L	1.91^**^	20	0.16	1.67	2.19
36–48 months	Female‐L	1.96	20	0.73	1.83	2.15
	Female‐L	1.99	20	0.78	1.81	2.13
	Male‐R	2.03	20	0.80	1.88	2.17
	Male‐L	2.01	20	0.82	1.88	2.16

** Statistically significant at *p* < 0.001 level.

Statistically significant correlation was observed between carcass weight and total calcaneus and talus morphometric values. In the young individuals, carcass weight was correlated with: GLc negatively at *p* < 0.05 level, sig (two‐tailed) = −0.374; GLt positively at *p* < 0.001 level, sig (two‐tailed) = 670; Calfactor positively at *p* < 0.001 level, sig (two‐tailed) = 965; Talfactor positively at *p* < 0.001 level, sig (two‐tailed) = 813; GLc/GLt ratio negatively at *p* < 0.001 level, sig (two‐tailed) = −508. No correlation was observed between carcass weight and GB, Bd, Calfactor and Talfactor values. In the adult individuals, carcass weight was correlated with: Calfactor positively at *p* < 0.001 level, sig (two‐tailed) = 861 and Talfactor positively at *p *< 0.001 level, sig (two‐tailed) = 873. No correlation was observed between carcass weight and GLc, GB, GLt, Bd, Calfactor and Talfactor values.

The constant term and coefficient of the regression equation were calculated for the parameters that were statistically significant. This equation was verified according to carcass weight. Accuracy rate is calculated as 85%. The equation that predicts carcass weight with Calfactor of adult male was given by the following equation:

*y* = 9.54 + 50.62*x*

## 4 DISCUSSION

Models for estimating body weight via physical characteristics are commonly used, especially in rural areas, and in cases where there is no access to scales, certain body measurements have great importance in determining body weight for farmers (Şengül et al., 2020; Mathapo et al., 2022). In addition, several algorithms such as data mining algorithms were developed for a physical characteristic that can be used to predict important economically valuable traits such as body weight for livestock (Eyduran, 2016). In the field of animal husbandry, the live weight of livestock is a crucial economic characteristic, and accurately forecasting it aids producers in making optimal decisions regarding animal breeding, allocating appropriate feed quantities, providing medical care and setting fair market prices for farm animals (Eyduran, 2016; Mathapo et al., 2022). However, it is believed that morphometric measurements of certain structures that comprise the skeleton, akin to the physical features used in estimating live weight, can be utilized in the prediction of carcass weight. In the present study, for this purpose, it was observed that certain morphometric measurements have a statistically positive correlation with carcass weight in predicting carcass weight, such as GLt, Calfactor and Talfactor.

On the other hand, the primary objective in animal breeding is to identify and utilize the most outstanding breeders to enhance the genetic quality of the stock effectively. For this purpose, researchers seek to develop new factors effecting selection strategies. In this regard, different data mining algorithms on the estimating of live body weight, such as chi‐squared automatic interaction detector, exhausted chi‐squared automatic interaction detector, classification and regression trees, Pearson correlation coefficient, coefficient of variation %, SD ratio, root‐mean‐square error, mean error, global relative approximation error, mean absolute percentage error, mean absolute deviation, coefficient of determination, adjusted coefficient of determination, Akaike information criterion, adjusted AIC linear regression, multivariate regression and LSM methods (Bassano et al., 2003; Dudhe et al., 2015; Roy et al., 2008; Tatlıyer Tunaz, 2021). According to the simple regression equations obtained from the current study, regression models seem to be an 85% valid method for estimating carcass weight. It is believed that the factors mentioned above can be utilized in determining the carcass weight by using the specified methods.

Results of the current study showed that age influenced all the morphometric measures studied, such that as the goat ages, the values of these traits also increase until 36–48 months old. These results agree with the findings of Idrissou et al. (2017) and Ofori et al. (2020), who found a highly statistical significant correlation between age and morphometric measures.

The relationship between age and weight gain in cattle fattening is extremely important. The fastest weight gain in cattle occurs at a young age. In this regard, calves come to the forefront. During the fattening period, although calves reach almost twice their initial fattening weight, the increase in 1‐year‐olds is 70%, and in 2‐year‐olds, it decreases up to 40%–50% (Ardıclı, 2019). The present study showed that hair goat weight gain is important in the first year after birth by comparing the mean values of carcasses of both young and adult goats.

According to Ardıclı, the physical structure of meat is generally characterized as meat, fat and bone. The ratios of these three substances to each other change with the age of the animal. There is a positive relationship between age and carcass bone ratio. In other words, increase in the age causes an increase in the bone composition of carcass (Ardıclı, 2019). In line with the current study's results, it has been identified that ageing leads to an increase in the carcass's bone ratio, consistent with Ardıclı’s research. Because the present results show strongly positive correlations between carcass weight and length of calcaneus and talus, breadth of talus as well as the factors calculated by morphometric measurements of these bones, changes in GLc, GLt, Bd, Calfactor and Talfactor could be taken into consideration in monitoring carcass weight.

In the Bassano et al. study for male ibex, the most accurate yearly body weight prediction was achieved using the square of the chest girth and age as predictor variables. In the case of females, age, total body length and wither height were the most effective predictors. Age was consistently chosen as a predictor variable in the prediction models (Bassano et al., 2003). Based on this point of view, carcass weight could be an indicator of growth performance, and in some cases, they are strongly correlated with each other. The present study supports the idea that age is a predictor of carcass weight, and morphometric measurements of talus and calcaneus may be an indicator of carcass weight by ageing.

In all domestic animals, the determination of live weight is crucial for both management and veterinary purposes. Although the most precise way to determine an animal's live weight is through weighing, this method is often impractical, particularly for larger animals, due to the lack of readily available scales. Furthermore, the process of weighing can be time‐consuming, potentially hazardous and may cause stress to the animal. In such situations, animal owners and veterinarians typically resort to estimating the animal's live weight based on visual evaluation and their personal experience. The obtained data from the current study may play an important role in designing correlations based on morphometric measurements of certain body parts and both live body weight and carcass weight. According to the present literature, many researchers have designed regression analyses to predict body weights or carcass weights and used many body measurements as an indicator (Bassano et al., 2003; Bene et al., 2007; Cam et al., 2010; Ofori et al., 2020; Rashijane et al., 2023). In designing new regression analyses, GLc, GLt, Bd, Calfactor and Talfactor could be reliable parameters to reflect reality.

Rashijane et al. applied a multivariate adaptive regression spline algorithm test to estimate the live body weights in Savannah goats. By looking at the body characteristics of hair goats, meat yield is correlated with live body weight and can be increased with selection (Rashijane et al., 2023; Tozlu Çelik & Oflaz, 2017). Selection studies based on the physical characteristics of hair goats can lead to an enhancement in meat production. This selection may be powerful with new regression analysis such as Rashijane et al. studied, but body characteristics should be replaced with morphometric measurements, indices or factors. The present study suggested that GLc, GLt, Bd, Calfactor and Talfactor can be strong estimators in new regression analyses with their significant correlations with age and carcass weight.

On the other hand, body weight and morphometric measurements are economically important traits for the genetic improvement of meat‐type goats in genetic studies, and it was observed that more than half of the highly significant genes influencing growth are in terms of age‐adjusted body weight and morphometric measurements (Moaeen‐Ud‐Din et al., 2022). Anatomical measurements, indices and factors can be used for comparing results of genetic studies. According to the current study, both young and adult female and male goats may be used in genetic selection to improve carcass weight studies by looking at their GLc, GLt, Calfactor and Talfactor.

Lambe et al. discussed the use of computed tomography (CT) in commercial sheep breeding schemes to estimate carcass tissue weights and distribution. They also mentioned a strong negative correlation between CT‐measured muscle density and intra‐muscular fat content in different sheep breeds (Lambe et al., 2008). While examining carcass by imaging techniques in goats, the data obtained from the current study could be used for designing new calculations and ratios while estimating carcass weight by morphometric measurements.

The current study is a pioneer of identifying key body regions for predicting carcass weight. However, considering the challenges in accessing adult goat carcasses, particularly in Turkey, longer term and larger sampled projects could be designed.

## 5 CONCLUSION

It is recognized that macro‐environmental factors, such as genotype, sex, type of birth, feeding conditions, age, year of birth and maternal age, have an impact on an animal's body measurements, which are known to be a reflection of their growth performance. In this regard, not only average phenotypic traits like height at the withers, body length and chest girth but also average values of specific anatomical structures, such as certain bone measurements, such as talus and calcaneus could serve as indicators of growth performance as well as carcass weight performance. In addition, new anatomical factors and indices may be produced, and new regression methods may be applied with these new parameters to predict carcass weight. However, in new selection methods to increase the carcass weight.

## AUTHOR CONTRIBUTIONS

**Yasemin Üstündağ**: Data curation; formal analysis; investigation; methodology; project administration; software; supervision; writing—review and editing. **Mehmet Kartal**: Conceptualization; funding acquisition; resources; validation; visualization; writing—original draft. The authors are responsible for all components of this work.

## CONFLICT OF INTEREST STATEMENT

The authors declare the nonexistence of conflicts of interest.

## FUNDING INFORMATION

None.

### ETHICS STATEMENT

None.

### PEER REVIEW

The peer review history for this article is available at https://www.webofscience.com/api/gateway/wos/peer‐review/10.1002/vms3.1544.

## Data Availability

Data are available on request from the authors.
